# Functional Piezocrystal Characterisation under Varying Conditions

**DOI:** 10.3390/ma8125456

**Published:** 2015-12-02

**Authors:** Xiaochun Liao, Zhen Qiu, Tingyi Jiang, Muhammad R. Sadiq, Zhihong Huang, Christine E. M. Demore, Sandy Cochran

**Affiliations:** 1 Institute for Medical Science and Technology, University of Dundee, 1 Wurzburg Loan, Dundee DD2 1FD, UK; x.liao@dundee.ac.uk (X.L.); m.r.sadiq@dundee.ac.uk (M.R.S.); cdemore@ieee.org (C.E.M.D.); 2 School of Science and Engineering, University of Dundee, Dundee DD1 4HN, UK; t.y.jiang@dundee.ac.uk (T.J.); z.y.huang@dundee.ac.uk (Z.H.); 3 Department of Electronics and Electrical Engineering, University of Strathclyde, Glasgow G1 1XW, UK; zhen.qiu@strath.ac.uk; 4 School of Engineering, University of Glasgow, Glasgow G12 8QQ, UK; sandy.cochran@glasgow.ac.uk

**Keywords:** piezocrystal, piezoelectric characterisation, high power, high resolution, high stress field, high temperature field, high electric drive field, mode shape, thermal response

## Abstract

Piezocrystals, especially the relaxor-based ferroelectric crystals, have been subject to intense investigation and development within the past three decades, motivated by the performance advantages offered by their ultrahigh piezoelectric coefficients and higher electromechanical coupling coefficients than piezoceramics. Structural anisotropy of piezocrystals also provides opportunities for devices to operate in novel vibration modes, such as the *d_36_* face shear mode, with domain engineering and special crystal cuts. These piezocrystal characteristics contribute to their potential usage in a wide range of low- and high-power ultrasound applications. In such applications, conventional piezoelectric materials are presently subject to varying mechanical stress/pressure, temperature and electric field conditions. However, as observed previously, piezocrystal properties are significantly affected by a single such condition or a combination of conditions. Laboratory characterisation of the piezocrystal properties under these conditions is therefore essential to fully understand these materials and to allow electroacoustic transducer design in realistic scenarios. This will help to establish the extent to which these high performance piezocrystals can replace conventional piezoceramics in demanding applications. However, such characterisation requires specific experimental arrangements, examples of which are reported here, along with relevant results. The measurements include high frequency-resolution impedance spectroscopy with the piezocrystal material under mechanical stress 0–60 MPa, temperature 20–200 °C, high electric AC drive and DC bias. A laser Doppler vibrometer and infrared thermal camera are also integrated into the measurement system for vibration mode shape scanning and thermal conditioning with high AC drive. Three generations of piezocrystal have been tested: (I) binary, PMN-PT; (II) ternary, PIN-PMN-PT; and (III) doped ternary, Mn:PIN-PMN-PT. Utilising resonant mode analysis, variations in elastic, dielectric and piezoelectric constants and coupling coefficients have been analysed, and tests with thermal conditioning have been carried out to assess the stability of the piezocrystals under high power conditions.

## 1. Introduction

Piezocrystals of the relaxor-PT type such as (*x*)Pb(Mg_1/3_Nb_2/3_)O_3_-(1-*x*)PbTiO_3_ (PMN-PT), (*x*)Pb(In_1/2_Nb_1/2_)O_3_-(1-*x*-*y*)-Pb(Mg_1/3_Nb_2/3_)O_3_-(*y*)PbTiO_3_ (PIN-PMN-PT) and Mn-doped PIN-PMN-PT (Mn:PIN-PMN-PT), respectively termed Generations I, II and III, have demonstrated electrical, mechanical and piezoelectric properties that can be translated into higher electroacoustic transducer performance than can be achieved with conventional piezoceramics [1] and PMN-PT has now established itself as the material of choice in biomedical ultrasound transducers [2,3,4,5]. However, this application has relatively undemanding requirements in terms of average power and operation at elevated temperature and pressure because of the output power and temperature safety limitations imposed by medical applications, and this matches well with the limited coercive field, *E_c_*, mechanical quality factor, *Q_m_*, and ferroelectric rhombohedral-tetragonal (*F_RT_*) phase transition and Curie temperatures (*T_RT_* and *T_C_*) of PMN-PT. In contrast, there are numerous applications, for example in underwater sonar, the oil and gas industries, medical therapy and ultrasound-actuated tools, where high average power handling and elevated temperature and pressure may be encountered [6,7]. These applications have prompted research into PIN-PMN-PT and Mn:PIN-PMN-PT, the former now available commercially (e.g., CTG Advanced Materials, Bolingbroke, IL, USA) and the latter in small quantities for testing purposes (TRS Technologies, State College, PA, USA).

Almost all piezocrystal production is based on the Bridgman technique [8,9], in which individual boules are grown in Pt crucibles, typically of 4′′ diameter for commercial supply. Unlike piezoceramic, the production of which by extrusion permits continuous and close to uniform processing, piezocrystal boule growth carries the possibility of boule-to-boule variation and, more fundamentally, involves inherent compositional gradients in the PT fraction. Continuous growth has been under development for some time [10] to reduce this problem but still awaits commercial adoption. All these lead to greater variations in material properties during production than are typical of piezoceramic. In turn, there is greater demand for routine measurement of the characteristics of piezocrystal. For the design of many electroacoustic transducers, the full elastoelectric matrix is required [11]. This can be measured with a standard process [12] based on analysis of the complex electrical impedance spectra of several material samples, each supporting a single, primary ultrasonic mode. Because of the possibility of variation between samples and the difficulty in achieving self-consistency in measurements from different samples, alternative techniques have been developed using ultrasound transit time measurement and resonant ultrasound spectroscopy [13,14] to obtain the elastic parameters, with the results combined with electrical measurements to obtain the full elastoelectric matrix. In this way, the material properties can be obtained from just one sample. This technique has also been extended to measurement at elevated temperatures [14] but not yet at pressures above ambient.

Whilst the full elastoelectric matrix is necessary for detailed transducer design using contemporary computer-modelling and virtual prototyping tools such as finite element analysis (FEA, e.g., PZFlex, Weidlinger Associates Inc., Mountain View, CA, USA), it is also possible to gain significant insight into piezocrystal properties and behaviour through characterisation of specific sample configurations [15]. This approach is appropriate when new materials are in development, both piezocrystals and other types such as Pb-free piezoceramics [16]. Although only limited subsets of material properties can be obtained, they are nevertheless valuable in controlling material production, in estimation of likely transducer properties, and sometimes for transducer design using constrained models [17]. Crucially, this approach also allows the materials to be characterised under practical conditions of elevated temperature, above ambient uniaxial pressure, and at high electrical excitation levels, the latter having the potential to cause self-heating and to elicit the effects of coercive field limits. In addition, it is possible to explore behaviour relating to specific material properties, such as acoustic modes relating to the unusually high *d_31_*, *d_32_*, *d_15_* and *d_36_* coefficients of relevant piezocrystal cuts [1], which can have many practical applications.

To evaluate the suitability of piezocrystals for practical applications, characterisation is currently undertaken in both industrial and academic research in relation to performance, consistency and stability. In industry, a datasheet is often supplied on delivery, including parameters such as permittivity, ε*_ij_*, piezoelectric constants, *d_ij_*, elastic compliance constants, *s_ij_*, electromechanical coupling coefficients, *k_ij_,* and frequency constant, *N_ij_*. These parameters define the material’s functionality in a particular vibration mode and always in an ambient environment. By examining the parameters in the datasheets, consistency across different piezocrystal samples can be checked within a batch or between different batches. It is rare to check the piezocrystal stability under varying conditions in industrial production unless a new application specifically encourages collaboration with academics, e.g., to examine a newly designed composition or mode [18]. Collaboration between industry and academic research then motivates more detailed and comprehensive characterisation [19,20] and the possibility of new standards such as the one under development, under the auspices of the IEEE, for relaxor-based piezocrystal for transducer and actuator applications [21].

In academic research, performance characterisation frequently includes measurement of the full elastoelectric matrix to gain understanding of a material under test [22,23,24]. This full matrix characterisation can be combined with investigation of compositional variation, domain engineering and special cuts to explore possible ultrahigh piezoelectric constants and novel vibration modes [8,24,25,26,27]. In addition to the full elastoelectric matrix, some key parameters are also measured, including *k_ij_*, dielectric loss, tanδ*_e_*, and mechanical quality factor, *Q_m_*. For particular applications, a figure of merit (FOM) may also be calculated, such as *k_ij_^2^Q_m_* for active underwater transducers or *d_h_g_h_/*tanδ*_e_* for passive underwater hydrophones where *d_h_* and *g_h_* are the piezoelectric constants under hydrostatic conditions [1].

Stable piezocrystal performance intrinsically relies on phase stability and can thus deteriorate significantly during phase transitions caused by external conditions such as temperature, pressure and electrical excitation. Stability is always explained in relation to *E_c_*, *T_C_* and *T_RT_*, and can be quantified as a percentage variation resulting from applied conditions, e.g. ∆*d_ij_/d_ij_* [28,29]. Characterisation of piezocrystals under deliberately conditioned fields is used to mimic real applications; this is crucial to understand performance variation, to design piezocrystal transducers with FEA, and to apply piezocrystal devices in practice. Characterisation as a function of temperature is usually conducted in an oven or thermal chamber; in this study, we also report self-heating as a parameter in stability testing and characterisation. Such self-heating directly relates to the mechanisms of dielectric, piezoelectric and mechanical losses, the equations for which have been derived for the common length-extensional (LE), length-thickness-extensional (LTE) and thickness-shear (TS) modes in piezoceramics but not yet in piezocrystal [30,31]. Pressure can be applied hydrostatically or along specific axes. Whilst hydrostatic pressure may appear most relevant, many high power transducer designs for which piezocrystal is suitable, such as the Tonpilz type for underwater SONAR [32,33] and various ultrasonically-actuated devices [8], include a uniaxial prestress. The simple uniaxial configuration can thus generate valuable results [34] and is one focus of this paper. In practice, piezocrystal may be subject to combinations of temperature and pressure making the situation more complicated and performance stability worse, and there is thus a pressing need for experimental exploration of such situations.

This paper focuses on application-oriented characterisation of the three generations of piezocrystals, all grown conventionally with the Bridgman method. The relatively diverse measurement methods that have been employed are described in Section 2. These include electrical impedance spectroscopy with standard instrumentation, which allows high average-power excitation when configured appropriately. In Section 3, a functional characterisation system is introduced including environmental and excitation factors relating to potential passive and active applications respectively. Section 4 outlines measurement conditions and results. The use of standard measurement instrumentation is combined with environmental heating in the range from ambient temperature to approximately 200 °C and with the application of uniaxial pressure up to 60 MPa, consistent with full ocean depth in underwater SONAR systems. High-average power excitation is combined with thermal imaging and control techniques to allow specific piezocrystal temperatures to be set and examined. The use of laser Doppler vibrometry (LDV) is illustrated through the direct measurement of piezoelectric coefficients and comparison is made with the authors’ use of the Berlincourt method. Ultrasonic transit-time testing can also be utilised to obtain elastic properties. In Section 5, the resulting material characteristics are related to fundamental properties, including crystallographic phase transitions and changes in elastic, dielectric and piezoelectric properties, and comparisons with other measurement techniques are discussed. Conclusions are drawn in Section 6 and measurement avenues open to further exploration are considered.

## 2. Characterisation Methods for Piezoelectric Materials

### 2.1. Material Properties and Measurement Methods

The translation of microstructural characteristics of piezocrystals such as crystal phase, domain configuration and polarization dynamics into macroscopic properties determines functional performance, with the macroscopic properties mathematically represented by material properties in tensor form. Using reduced notation, these are collated in matrices which can take multiple forms according to convenience. Here, we assume the full elastoelectric matrix contains elastic (stiffness), dielectric and piezoelectric properties, *c_ij_*, ɛ*_ij_* and *d_ij_*. For functional evaluation, another two performance characteristics *k_ij_* and *Q_m_* are also often used. The measurement of all these characteristics can be made using: (1) the Berlincourt method; (2) electrical impedance spectroscopy; (3) LDV; and (4) ultrasonic methods. Not all four methods can characterise all the properties; compatibility between properties and the measurement methods is shown in Table 1.

**Table 1:** Compatibility between piezocrystal properties and measurement methods.

Properties	Measurement Methods			
	Berlincourt Method	Electrical Impedance Spectroscopy	Laser Doppler Vibrometry	Ultrasonic Methods
Dielectric Properties		√		
Piezoelectric Properties	√	√	√	
Elastic Properties		√		√
Electromechanical Coupling Coefficient		√	√	
Mechanical Quality Factor		√	√	

The *Berlincourt method* [35] measures the fundamental piezoelectric charge coefficient, *d_ij_*, based on the direct piezoelectric effect, when electrical charge is generated from applied mechanical force. During quasi-static measurement, the mechanical force is applied at a frequency lower than any resonance in the sample under test. The Berlincourt method is thus insensitive to sample geometry, as long as electrodes are present to make electrical contact. It can obtain comparable *d_33_* values from both a tall, narrow LE mode bar and from a thin thickness-extensional (TE) mode plate, if the two samples are both fully polarised. Variations of *d_33_* values in practical measurement may be induced by incomplete polarisation, as previously reported for PMN-PT piezocrystal plates with different thicknesses [36]. Commercial *d_33_* meters provide a fast, convenient way to measure *d_31_* and *d_15_* values with specially designed fixtures (e.g., ZJ-6B, CTG Advanced Materials, Bolingbroke, IL, USA).

*Electrical impedance spectroscopy* of a piezocrystal sample is achieved by applying an AC signal with frequency swept over a selected range. The current amplitude and phase are recorded and the complex impedance is obtained with reference to the driving voltage [37]. Based on the impedance spectrum, electrical and mechanical resonances and their corresponding frequencies can be identified, allowing evaluation of the piezoelectric and elastic properties strongly coupled to particular resonant modes. Many commercial impedance analysers are available, but the same technique can also be realised with less specialised instrumentation, as we describe later for high power characterisation. Methods of analysis of electrical impedance spectra in the IEEE standard [12] specify multiple resonant geometries with poling directions and recommended aspect ratios to ensure the target resonant behaviour is the main motion in the specified direction, well separated from any others in frequency. Based on the resonance frequencies of these samples, all the fundamental piezoelectric constants can be calculated. However, this requires multiple samples which can lead to inconsistency in piezocrystal characterisation.

LDV is a direct, non-invasive way to measure *d_ij_* based on the inverse piezoelectric effect. In this method, the LDV measures the surface displacement when a non-resonant electrical field is applied across the electrodes of a given sample; *d_ij_* can be calculated from the measured displacement, applied voltage and sample geometry. The vibration boundary conditions must be strictly respected by mounting the sample on a nodal plane and the measurement frequency should differ from any resonance in the sample to avoid the interference from the effect of *Q_m_*. LDV can also be used with frequency sweeping across a resonance to evaluate both *k_ij_* and *Q_m_*.

A simple ultrasonic method based on the pulse-echo technique can be used to measure the elastic properties of solid materials. The full set of stiffness constants can be determined with longitudinal and shear waves applied in different orientations. It has been suggested that this method provides better accuracy in determining the elastic constants of piezoelectric material than the impedance spectroscopy method [38], but it has insufficient accuracy for the piezoelectric constants. A combined ultrasonic-resonance method has therefore been developed by Cao *et al.* [39], and this also reduces the number of samples required. Recently, the ultrasound method has been combined with inverse electrical impedance spectroscopy to determine the full elastoelectric property matrix from only one sample [13]. This technique guarantees self-consistency of the full property matrix by eliminating sample-to-sample variation.

### 2.2. Other Methods for Sample Size Reduction and Loss Mechanism Characterisation

Some other newly proposed characterisation methods for piezocrystals are in development. Drawing on previous work in geology and passive material characterization, resonant ultrasound spectroscopy (RUS) has been used to determine the elastic properties of a hard lead zirconate titanate (PZT) composition using one sample under different temperature conditions [14]. However, the single sample is usually a cube which RUS requires to be mounted on two body diagonal corners. Care must be taken to avoid damage during preloading, and the mounting method makes it difficult to test these samples under high stress conditions. Another method has been proposed based on loss mechanisms [30] using loss factor equations derived for the modes associated with *k_33_*, *k_31_*, *k_15_* and *k_t_*. Based on these, the loss factors can be easily characterised for dielectric, piezoelectric and elastic losses rather than to measure the hysteresis between stress, strain, electric displacement and electric field. This method may help understanding of heating effects in piezoelectric transducers, but it has not yet been applied to piezocrystals.

### 2.3. Requirements of Piezocrystal Samples

**Figure 1:**
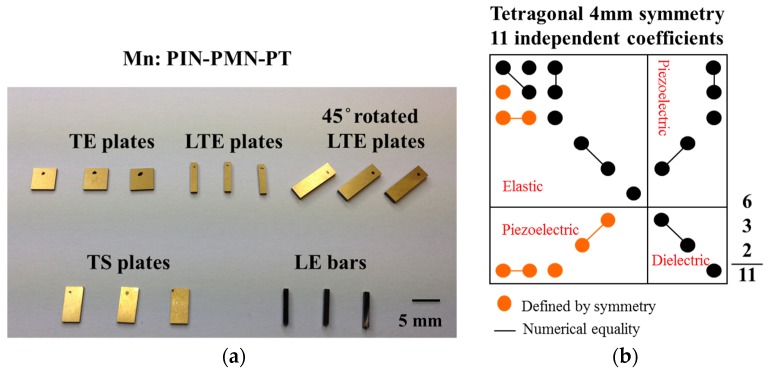
(**a**) Sample set of Mn:PIN-PMN-PT for full elastoelectric matrix; (**b**) Dependence of elastic, piezoelectric and dielectric constants for 4 mm symmetry in full elastoelectric matrix.

For many years, the IEEE standard method and its precursors [11] have been used to determine the full elastoelectric matrix. This requires a separate material sample with a specified geometry for each of several defined propagation modes, as shown in Figure 1. The number of samples differs according to the crystallographic symmetry of the material system to be characterised. The (001)-poled piezocrystal has 4 mm symmetry and therefore requires five samples, operating in the TE, TS, LE and LTE modes, with the latter also required 45°-rotated. Two of these samples raise particular issues. The TS sample must be poled in one direction then have the poling electrodes removed and replaced on a different pair of surfaces; none of the other samples requires this preparation. The LE sample is specified to have a width:thickness aspect ratio of 1:10. Because of the compositional gradient along the length of a piezocrystal boule, combined with a relatively short section with properties that are acceptable in terms of the performance of electroacoustic transducers, even a lateral cross-section as small as 1 mm square, from which a 10 mm long LE bar is cut, may result in significant compositional variation within the sample. In addition, long samples can elicit problems in poling and samples of small cross-section have high impedance. The specified aspect ratio for the LE bar is therefore sometimes adjusted, e.g., to 1:5 or in the authors’ experience and as published [40,41] as low as 1:3, provided that unimodal LE operation is observed explicitly.

Given the variation in material properties along a crystal boule and potential variations in poling, the use of multiple samples to provide subsets of the elastoelectric matrix raises the possibility of inconsistency in the matrix and this has been found to be the case in practice [15]. Therefore, the alternative methods outlined previously are under development, with the aim to reduce the number of samples required [13,14]. However, such methods leave two issues unresolved. First, it would be very unusual to use a multimodal piezoelectric material configuration in a practical electroacoustic transducer, as the intention is usually to base such transducers on unimodal configurations to avoid performance degradation by cross-coupling of the modes. Thus, the single sample on which characterisation is based is likely to differ from the configurations used in practical transducers with consistency of poling requiring particular attention. Second, compositional variation along the length of the boule and potential variation through insufficiently controlled growth mean that measurement even of a single sample does not represent the whole boule. Nevertheless, single sample characterisation overcomes difficulties with self-consistency because of sample-to-sample variation and is thus advantageous compared with the standard method.

An alternative approach is to obtain incomplete but useful measurements from selected material configurations. For example, for the many electroacoustic transducers utilising the TE mode, very useful information can be gained by characterising TE mode samples and the same principle applies for other modes. Since most transducers intentionally exploit only one mode, a great deal of value can be gained from such characterisation in estimating transducer performance, checking sample-to-sample uniformity and elucidating behaviour under different conditions of temperature and pressure. This approach is the one taken here with measurements illustrated in the following sections based on TE, LTE and novel *d_36_* face shear (FS) mode behaviours. In this context, the samples on which most results in this paper are based are TE mode with typical thickness of 0.5 mm and essentially unimodal behaviour achieved over the fundamental ultrasonic frequency range by meeting the constraint of a width:thickness ratio of 10:1. Thus, most samples have dimensions 5 mm × 5 mm × 0.5 mm. Furthermore, a representative range of properties is often used. Here, we focus on the dielectric coefficient, ε*_r33_^S^*, the stiffness, *c_33_^D^*, the piezoelectric coefficient, *e_33_*, and the electromechanical coupling coefficient, *k_t_*, as these provide useful information across a wide range of materials and applications.

## 3. Piezoelectric Functional Characterisation System

**Figure 2:**
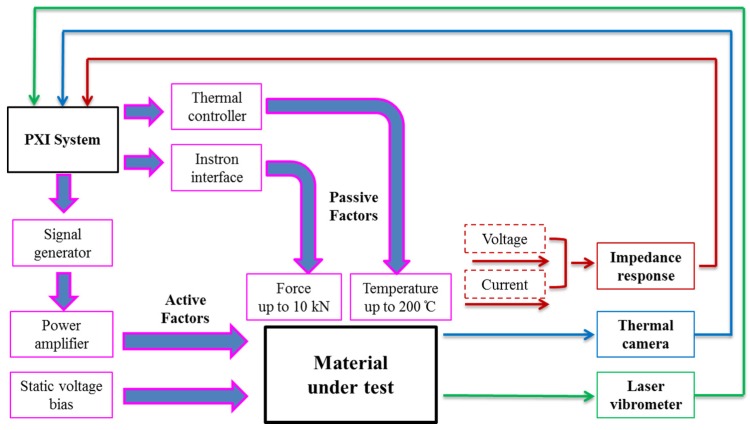
Piezoelectric functional characterisation system diagram.

**Figure 3:**
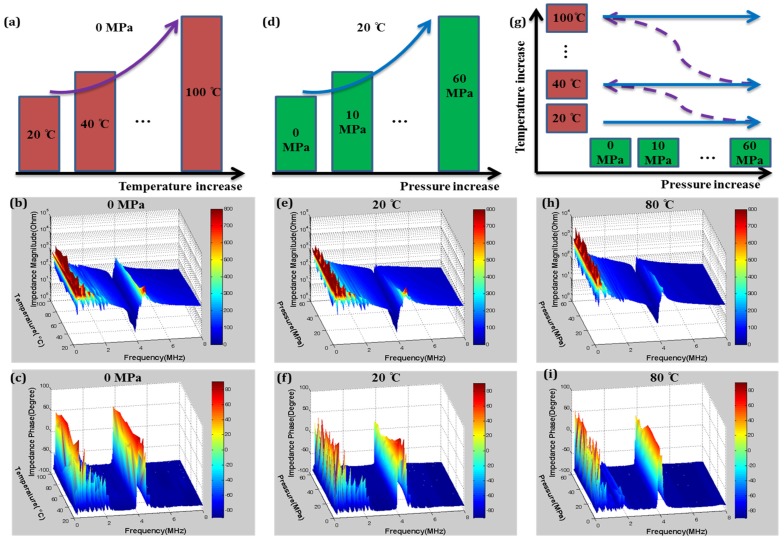
Characterisation of Mn:PIN-PMN-PT: (**a**) Configuration for variation of temperature at pressure of 0 MPa with measurement of (**b**) impedance magnitude and (**c**) impedance phase; (**d**) Characterisation configuration for variation in pressure at ambient temperature with measurement of (**e**) impedance magnitude and (**f**) impedance phase; (**g**) Characterisation configuration for variation in temperature and pressure with measurement of (**h**) impedance magnitude and (**i**) impedance phase.

The system on which the work reported here is based is shown schematically in Figure 2. Key components are an electrical impedance analyser (e.g., 4395A, Agilent/Keysight, Santa Clara, CA, USA) and a material testing system (MTS, SSID 5966, Instron Ltd., High Wycombe, UK) with which a bespoke, fan-assisted oven is integrated, allowing penetration of the roof and floor by the MTS applicator bars. These instruments are controlled with a PXI-based PC running software implemented in LabVIEW (National Instruments, Houston, TX, USA). This software is able to select a particular pressure profile with multiple levels in the range 0–60 MPa and simultaneously control the oven temperature in the range from ambient to 200 °C. Data can be acquired over multiple contiguous frequency ranges, thus extending the number of sampling points arbitrarily beyond the standard limit of 801 points from the impedance analyser, consequently enhancing the measurement resolution. In practice, the system is operated in a mode in which a given temperature is set, starting with the lowest temperature required, then the pressure is incremented, applied uniaxially with brass contact pads dry-coupled to the sample under test to minimise energy leakage from it. After the pressure has reached the specified maximum and the first set of measurements has been completed, the pressure is reduced to the minimum, the temperature is increased to the next set level, and the pressure cycle is repeated, as shown in Figure 3g. Dependency of material properties on previous conditions must thus be considered in the analysis of the results, which is based on calculation of material properties from resonant frequencies observed in the impedance spectra. This basic configuration focuses on the environmental factors of elevated temperature and pressure, targeting passive piezocrystal applications.

For characterisation of piezocrystals under active application conditions, the basic system is complemented by other measurement capabilities. If necessary, and of particular relevance to PMN-PT because of *E_c_* values in the range 1–2 kV·mm^−1^, an electrical field bias can be applied with a high voltage power supply. For the TE samples defined previously, *V* = 1 kV is sufficient for many measurements. A scanning LDV can provide information about modal behaviour and as noted previously, can be used directly to determine the piezoelectric modulus through a few single-point measurements of displacement of the relevant surface of the sample under off-resonant excitation. It is also possible to measure electrical impedance at high average-power excitation levels by driving the sample under test with a power amplifier and determining voltage, current and their relative phase with appropriate probes. This technique has been taken further by observing heating of the sample under test with a thermal imaging camera (TIM-160, Micro-Epsilon Ltd., Ortenburg, Germany). This self-heating measurement provides information on both temporal thermal behaviour and impedance spectral response at a set temperature achieved by controlling the applied voltage and current. In summary, the material characterisation setup, described above, can be used experimentally to characterise piezocrystal material in both passive and active electroacoustic transduction conditions.

## 4. Piezocrystal Characterisation

Based on the designed piezoelectric functional characterisation system, measurements were performed on three generations of piezocrystals. Most measurements were carried out on Generation II and III, 33% PIN-PMN-PT and Mn:PIN-PMN-PT samples, respectively, while we also include measurement results on PMN-29% PT samples from the literature [15] where they provide useful data for this paper. Effects of increased environmental temperature and pressure were investigated under passive conditions, *i.e.* with the piezocrystal samples un-driven. Electric field effects were determined with the piezocrystal driven at various voltages. In addition, LDV was used under ambient conditions to measure the piezoelectric constants *d_33_*, *d_31_*, *d_15_* and *d_36_*, with the results compared with the values obtained by the Berlincourt method and electrical impedance spectroscopy. Although only applied to Mn:PIN-PMN-PT, this is sufficient to demonstrate characterisation with LDV.

### 4.1. Environmental Temperature and Pressure Effects

#### 4.1.1. Impedance Spectra with Elevated Temperature and Pressure

As an illustration of our passive characterization methods, Figure 3 presents electrical impedance spectra of a 5 mm × 5 mm × 0.5 mm TE mode plate of Mn:PIN-PMN-PT with varying temperature, *T*, and pressure, *P*. Although rarely found in the materials literature, this is a familiar data presentation format for transducer developers evaluating material and device performance. The frequency ranges for these graphs were chosen to centre the TE-mode resonance with lateral resonances also appearing at much lower frequencies. Although these minor modes are not analysed further here, they provide clear evidence of behavioral changes with environmental conditions. As described previously, temperature and uniaxial compressive stress were applied simultaneously to the sample, following a pre-programmed testing protocol. Although impedance spectra were recorded for every condition and further analysed in later sections, only selected results are presented here: Figure 3a–c present results for 20 °C ≤ *T* ≤ 100 °C with *P* ambient; similarly, Figure 3d–f present results for 0 MPa ≤ *P* ≤ 60 MPa at ambient temperature; and Figure 3g–i show the combined effects of *T* and *P*, with *T* = 80 °C and 0 MPa ≤ *P* ≤ 60 MPa.

As expected, Mn:PIN-PMN-PT is relatively stable over the temperature range up to 100 °C which is most important for applications and which was used for the measurements shown in Figure 3, taking into account that *T_RT_* > 120 °C. The resonance frequencies and magnitudes for the minor lateral resonances and the main TE resonance decrease as only *T* increases. Similarly, the application of only *P* impedes vibration in both the planar and thickness directions, manifesting as decreases in the resonance frequencies and magnitudes; the effect of pressure is clear even at levels as low as *P* = 5 MPa. Combined *T* and *P* have increased influence on piezoelectric behaviour, with a significant decrease and then a slight recovery for *P* > 40 MPa, as seen in Figure 3i.

#### 4.1.2. Property Variation with Environmental Temperature

Using the IEEE resonance method, it is possible to extract specific material properties from the data presented in Figure 3. With the TE mode plates under test, ε*_r33_^S^, k_t_*, *e_33_* and *c_33_^D^* can be derived from:

(1) $${\varepsilon_{r33}}^{S}=t\cdot C/\left(A\cdot \varepsilon_{o}\right)$$

(2) $$k_{t}=\sqrt{\;\frac{\pi f_{s}}{2f_{p}}\tan\left(\frac{\pi }{2}\cdot \frac{f_{p}-f_{s}}{f_{p}}\right)}$$

(3) $$c_{33}^{D}=4\rho t^{2}{f_{p}}^{2}$$

(4) $$e_{33}=\;k_{t}\sqrt{\;{\varepsilon_{33}}^{S}\cdot c_{33}^{D}}$$

where *t* is sample thickness, ρ is density and *A* is the lateral surface area of the sample plate. *C* is the capacitance measured at a frequency double the sample’s mechanical resonance frequency; *f_p_* is the equivalent circuit parallel resonance frequency measured at peak electrical impedance magnitude, and *f_s_* is the equivalent circuit series resonance frequency measured at minimum electrical impedance magnitude.

**Figure 4:**
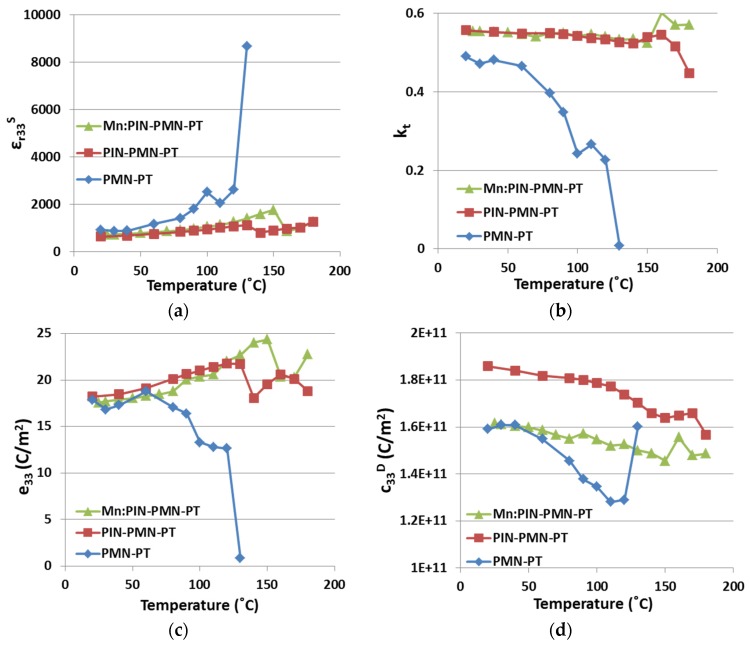
The effect of *T* on three generations of piezocrystals (**a**) ε*_r33_^S^*; (**b**) *k_t_*; (**c**) *e_33_*; and (**d**) *c_33_^D^*. (PMN-PT results adapted from [15]).

Figure 4 presents the variations in the four selected piezoelectric properties with elevated environmental temperature for all three generations of piezocrystals. These measurements were conducted in the range 20 °C ≤ *T* ≤ 180 °C. For PMN-PT, for which the expected range for use in applications is approximately 20–80 °C, ε*_r33_^S^* shows reasonable uniformity up to *T*~80 °C. A perturbation is then observed in the interval 90 °C < *T* < 120 °C, corresponding with the phase transition zone [42]. With temperature increasing towards 130 °C, ε*_r33_^S^* experiences a significant increase which indicates the material is depoled and piezoelectric behaviour disappears. The same trends are observed for *k_t_*, *e_33_* and *c_33_^D^*. In comparison, PIN-PMN-PT maintains piezoelectric behavior and is stable for *T* ≤ 120 °C. A phase transition then occurs in the range 120 °C < *T* < 160 °C, about 30 °C higher than for PMN-PT. For *T* > 160 °C, the piezoelectric behaviour deteriorates significantly, as expected when approaching *T_C_*, reported at around 180 °C [43]. Mn:PIN-PMN-PT is expected to be less temperature dependent than PIN-PMN-PT and PMN-PT because of the presence of Mn dopant [25]. In Figure 4, the phase transition of Mn:PIN-PMN-PT is shown in the range 140 °C < *T* < 170 °C, and *T_C_* is not approached at or below 180 °C, as expected from the literature [27]. Table 2 lists the variations of the four properties in details, over the range of 20 °C ≤ *T* ≤ 80 °C before PMN-PT piezocrystal experiences the *F_RT_* phase transition.

**Table 2:** Variation with *T* for three generations of piezocrystals, 20°C ≤ *T* ≤ 80°C (Reference *T* = 20°C).

(20 °C < *T* < 80 °C)	ε*_r33_^S^* (%)	*k_t_* (%)	*e_33_* (%)	*c_33_^D^* (%)
PMN-PT	+53.7	−19.2	+5.2, −5.9	+1.1, −8.5
PIN-PMN-PT	+29.0	−1.4	+10.4	−2.7
Mn:PIN-PMN-PT	+26.7	−1.1	+6.9	−4.1

+: maximum variation above the reference; −: minimum variation below the reference.

#### 4.1.3. Property Variation with Uniaxial Pressure

**Figure 5:**
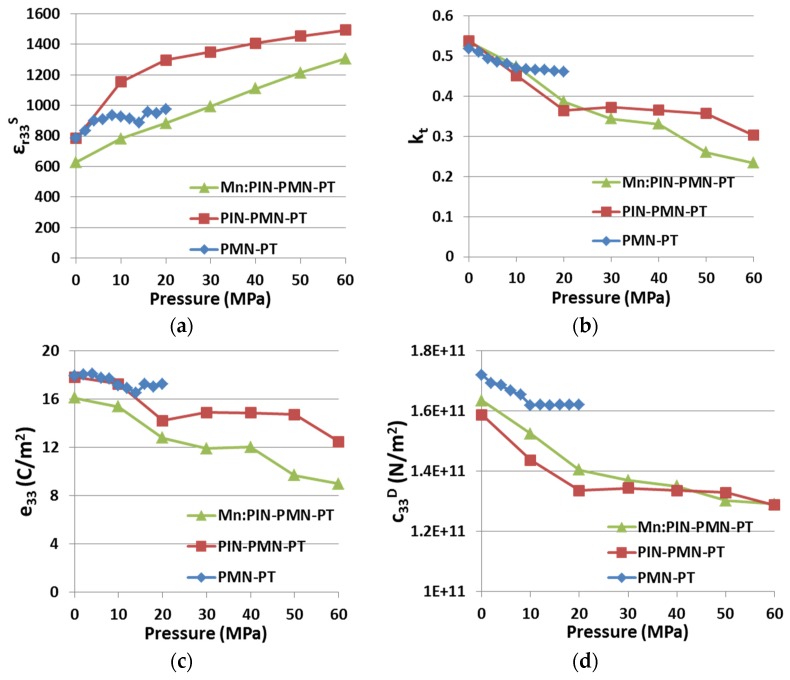
Effect of *P* on three generations of piezocrystals (**a**) ε*_r33_^S^*; (**b**) *k_t_*; (**c**) *e_33_*; and (**d**) *c_33_^D^*. (PMN-PT results adapted from [15]).

Figure 5 presents the variations of ε*_r33_^S^, k_t_*, *e_33_* and *c_33_^D^* with applied uniaxial pressure for all three generations of piezocrystals. The results presented for PIN-PMN-PT and Mn:PIN-PMN-PT piezocrystal were obtained using the automatic system described in Section 3 and the results for PMN-PT are taken from our previous work for comparison. Considering the range 0 MPa ≤ *P* ≤ 20 MPa under which the PMN-PT sample was tested, *P* appears to affect PMN-PT material relatively little compared with the other two materials. This may be because different test fixtures were used to contact the samples in these two sets of experiments.

Despite the differences in the magnitudes of the variations for the three generations, the trends are in reasonable agreement. For PMN-PT, perturbations are evident for 10 MPa ≤ *P* ≤ 14 MPa, which may be termed the phase transition zone induced by uniaxial compressive pressure. For PIN-PMN-PT, this pressure range is extended to around 20 MPa. For the Mn:PIN-PMN-PT sample, however, there is no obvious evidence of a phase transition zone. This suggests that the Mn acceptor dopant has significantly hardened the material to allow it to work under more demanding conditions than can be sustained with Generations I (PMN-PT) and II (PIN-PMN-PT) piezocrystals.

Details of the variations between PIN-PMN-PT and Mn:PIN-PMN-PT are listed in Table 3, with pressure ranging from 0 to 60 MPa. The variations of PMN-PT are not included because of the different, more limited pressure test range, *P* < 20 MPa, to which it was subjected.

**Table 3:** Variation with *P* for two generations of piezocrystals, 0 MPa ≤ *P* ≤ 60 MPa (Reference *P* = 0 MPa).

(0 MPa < *P* < 60 MPa)	ε*_r33_^S^* (%)	*k_t_* (%)	*e_33_* (%)	*c_33_^D^* (%)
PIN-PMN-PT	+90.8	−43.7	−29.9	−18.9
Mn:PIN-PMN-PT	+107.8	−56.4	−44.2	−21.0

+: maximum variation above the reference; −: minimum variation below the reference.

#### 4.1.4. Property Variation with Environmental Temperature and Uniaxial Pressure

**Figure 6:**
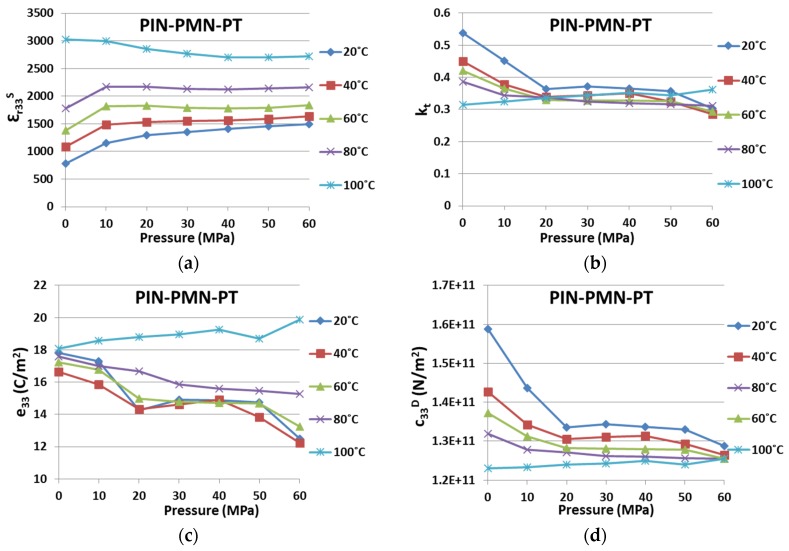
Property variation caused by environmental temperature and uniaxial pressure for PIN-PMN-PT on (**a**) ε*_r33_^S^*; (**b**) *k_t_*; (**c**) *e_33_*; and (**d**) *c_33_^D^*.

**Figure 7:**
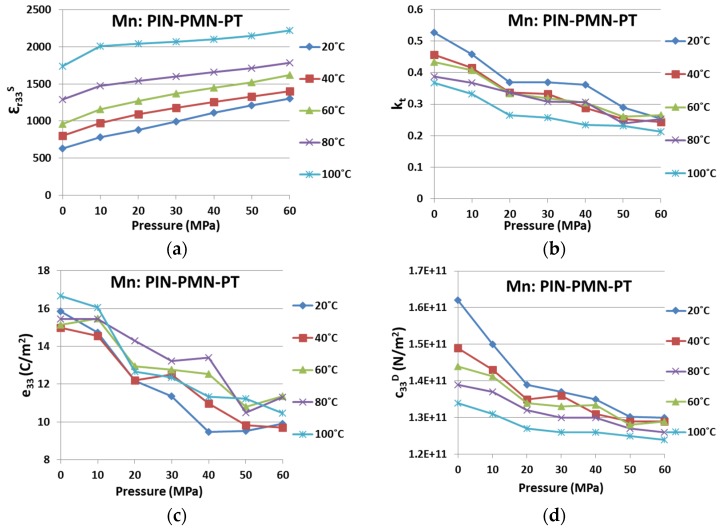
Property variation caused by environmental temperature and uniaxial pressure for Mn:PIN-PMN-PT on (**a**) ε*_r33_^S^*; (**b**) *k_t_*; (**c**) *e_33_*; and (**d**) *c_33_^D^*.

To look at the dual influences from environmental temperature and uniaxial pressure simultaneously, Figure 6 and Figure 7 present the variations for the four key piezoelectric properties in PIN-PMN-PT and Mn:PIN-PMN-PT, respectively. Variations in the characteristics are shown with environmental conditions of *T* and *P*, for 20 °C ≤ *T* ≤ 100 °C with a step of 20 °C and for 0 ≤ *P* ≤ 60 MPa with a step of 10 MPa.

As illustrated in Figure 6, for 20 °C ≤ *T* ≤ 80 °C, both *k_t_* and *c^D^_33_* of PIN-PMN-PT decrease with *P*, while ε*_r33_^S^*, on the contrary, increases with increasing *T* and *P*, and *e_33_* decreases with *P* and increases with *T* in general. At *T* = 100 °C, the opposite trend was observed, with *k_t_, c^D^_33_* and *e_33_* rising, and ε*_r33_^S^* falling in value following increased *P*. It is believed that the combined effect of applied cycles of *P* and increases in *T* has induced a phase transition at lower temperature, with the piezocrystal transferring from the rhombohedral to orthorhombic phase, as suggested by McLaughlin [41].

In comparison, Mn:PIN-PMN-PT in Figure 7 behaves similarly to PIN-PMN-PT, except that the same general trends are maintained for all four properties, even at *T* = 100°C. Again, this provides evidences that Generation III piezocrystals are more resilient to *T* and *P*, and thus likely to be more suitable for high power applications. However, as indicated in Table 3 and Table 6, the absolute variations in Mn:PIN-PMN-PT are bigger than for PIN-PMN-PT. This is because the deteriorating trends shown for Mn:PIN-PMN-PT with *T* and *P,* especially with *P*, did not recover after the phase transition.

### 4.2. Electric Field Induced Temperature Rise

In high power applications such as active SONAR, conventional piezoelectric materials are subject to high cyclic electric fields to generate large displacement outputs. In turn, the intrinsic mechanical, piezoelectric and dielectric losses generate considerable heat, leading to an increase in *T* and consequently affecting the material’s phase structure, piezoelectric properties and performance stability. This performance deterioration is exacerbated when the cyclic electric field is applied continuously for long periods of time, especially in industrial applications, such as ultrasonic cleaning, cutting and welding. In the context of integration of piezocrystals in such applications, it is necessary to carefully investigate these materials at different operating temperatures, and hence drive voltages.

**Figure 8:**
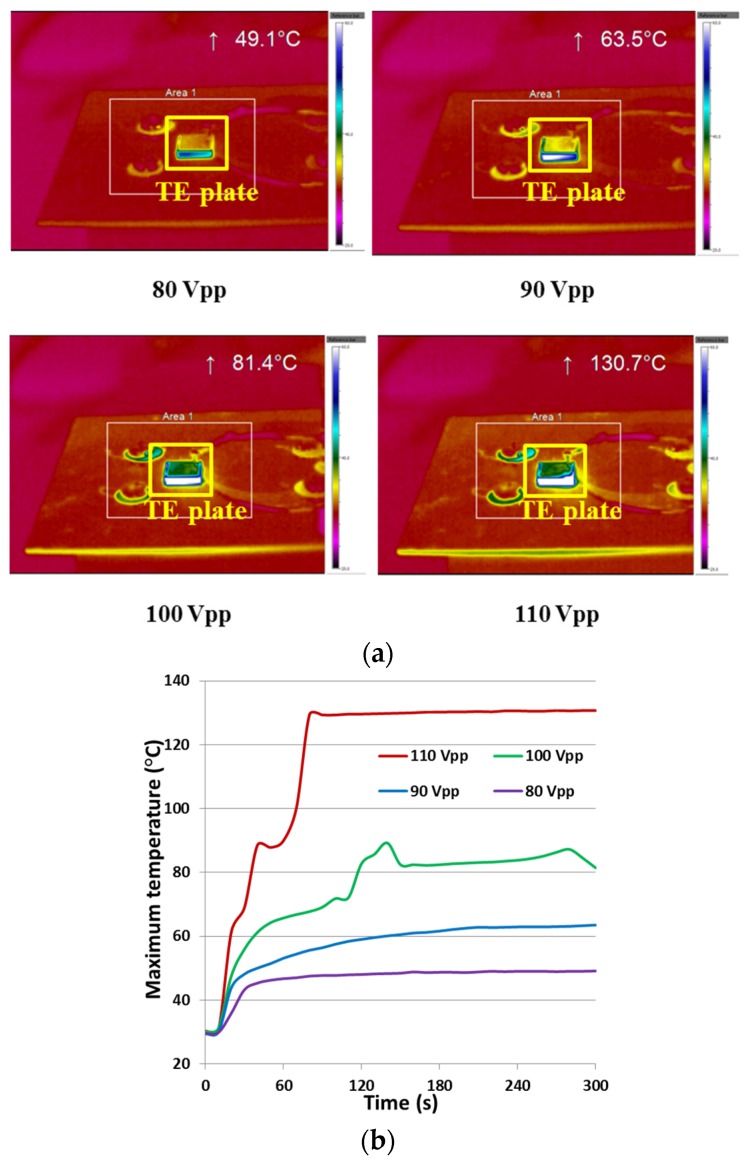
(**a**) Thermal mapping and (**b**) Maximum spot temperature of a PMN-PT TE plate driven with different voltages for 5 min.

Both temporal and spectral responses were examined for performance assessment. First, characterisation of the temporal response was conducted on a 10 mm × 10 mm × 1 mm PMN-PT TE plate, with AC driving voltages of 80, 90, 100 and 110 *V_pp_*, with an RF power amplifier (31002A, Electronics & Innovation, Rochester, NY, USA). The driving voltage was set at the plate’s resonant frequency of 2.19 MHz, and was applied continuously for 5 min. The thermal response was recorded with a thermal imaging camera (TIM-160, Micro-Epsilon Ltd., Ortenburg, Germany), as shown in Figure 8.

From the thermal maps of the TE plate sample in Figure 8a, it can be seen that the maximum electric field-induced temperatures were 49.1, 63.5, 81.4 and 130.7 °C respectively for 80 *V_pp_*, 90 *V_pp_*, 100 *V_pp_*, and 110 *V_pp_* sinusoidal excitation for 5 min. The temporal response curves in Figure 8b show that at 80 *V_pp_* and 90 *V_pp_,* *T* rose monotonically and then stabilised; however, at 100 and 110 *V_pp_*, *T* experienced particularly sharp increases at 110 s and at 60 s, corresponding to temperatures of 68.9 and 96.9 °C respectively. These increases may indicate a phase transition, as reported recently as *T_RT_* = 97 °C for PMN-PT [42].

Subsequently, characterisation of electric field-induced temperature increases was performed with respect to the spectral response, measuring the electrical impedance of the samples at induced temperatures maintained at stable levels. This spectral characterisation was performed on PIN-PMN-PT and Mn:PIN-PMN-PT TE plates with dimensions 5 mm × 5 mm × 0.5 mm. The electric field-induced temperature was maintained at successive temperatures of 40, 60, 80, 100 and 120 °C with the plate driven at its TE resonance with the AC driving voltage controlled adaptively. Impedance spectra were measured in the range 10 Hz–10 MHz with 800 sampling points, using a 10:1 passive voltage probe (N2862B, Agilent Technologies, Santa Clara, CA, USA) and a current probe (P6021A, Tektronix, Plano, TX, USA). As with the use of commercial impedance analyser systems, fixture compensation was performed to ensure accurate measurement calibration.

**Figure 9:**
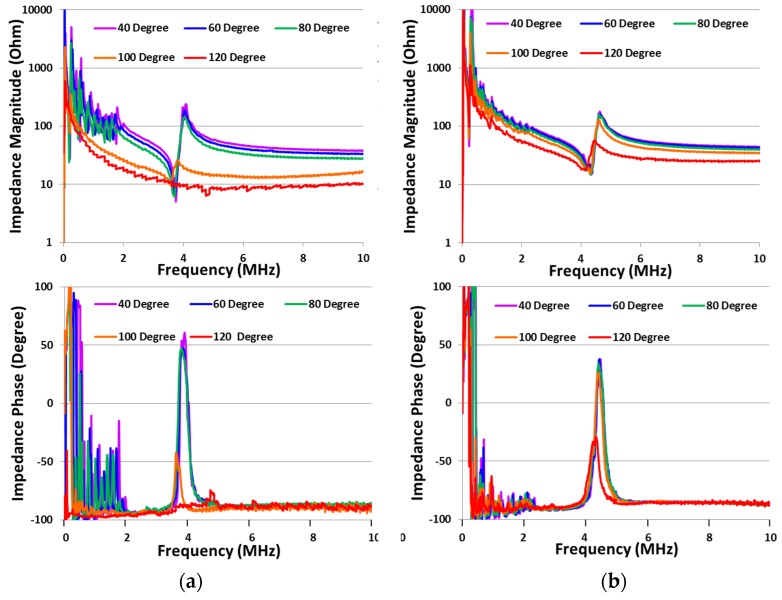
Impedance spectra of (**a**) PIN-PMN-PT and (**b**) Mn:PIN-PMN-PT when the electric field induced temperature was maintained as 40, 60, 80, 100 and 120 °C.

In Figure 9b, Mn:PIN-PMN-PT shows significant changes in the resonant impedance peak at 120 °C with an average driving voltage of 26 *V_pp_*. For PIN-PMN-PT in Figure 9a, the corresponding peak changes significantly at the lower temperature of 100 °C, with an average driving voltage of 28 *V_pp_*. These changes in impedance peaks may indicate the critical phase transition point for piezocrystals. For PIN-PMN-PT, the TE plate sample was depolarised at 120 °C with an average driving voltage of 84 *V_pp_*. The Mn:PIN-PMN-PT sample exhibited better thermal stability at high electric field than PIN-PMN-PT sample. It is also notable that *T* = 120 °C is far below *T_C_* (=191 °C [1]) for PIN-PMN-PT; the effects we observed may be a result of the combination of high *E* and *T*.

### 4.3. Laser Doppler Vibrometry under Ambient Conditions

LDV is based on the use of the inverse piezoelectric effect to measure sample surface vibration amplitude with applied electrical field. Not needing physical contact with the sample, LDV avoids the interference and energy loss from ultrasonic coupling at the interface that is unavoidable in most other measurements. It can be performed at a single measurement spot or by scanning a surface to obtain a mode shape.

**Figure 10:**
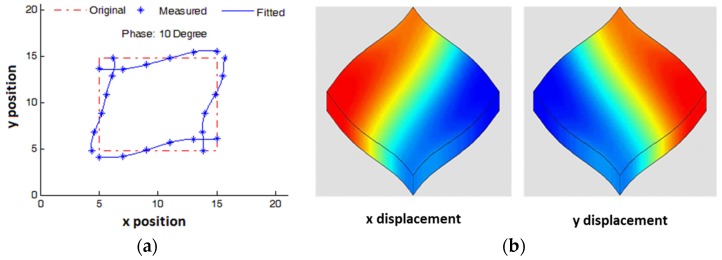
(**a**) Experimentally measured mode shape of a *d_36_* FS plate; and (**b**) corresponding simulated mode shapes with displacements along *x* and *y* directions mapped in colours.

In the work reported here, LDV was first used to measure the mode shape of a *d_36_* FS plate by scanning the four side surfaces around the plate, with both vibration amplitude and phase data acquired. Based on the measured data, the FS mode shape can be obtained by sweeping through the phases. In Figure 10a, the red dashed lines represent the original shape of the plate; the blue asterisks represent the LDV-measured vibration at 10° phase; the blue solid lines represent the curves fitted to the measured data points, illustrating the plate shape profile for FS vibration. The measured FS mode shape had a good agreement with the FS mode shape simulated with the PZFlex FEA software package, with displacements mapped along *x* and *y* directions, as shown in Figure 10b.

**Figure 11:**
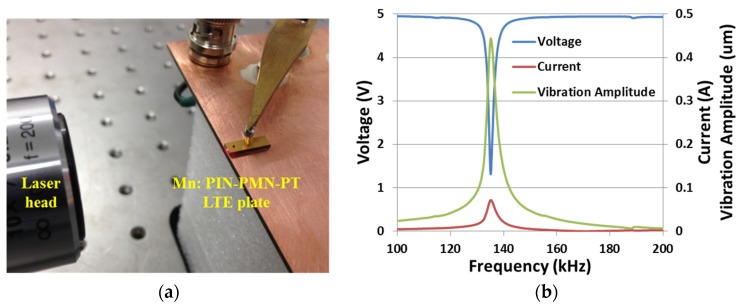
(**a**) Laser vibrometry characterisation setup with a Mn:PIN-PMN-PT LTE plate with (**b**) responses of voltage, current, and vibration amplitude when a constant AC 5 V driving voltage was swept from 100 to 200 kHz.

LDV was also used to measure piezoelectric constants, *d_ij_*. For a 10 mm × 3 mm × 1 mm Mn:PIN-PMN-PT LTE plate with 45° rotation about the *z* axis, the piezoelectric constant can be determined with:

(5) $$d_{31}=\frac{S_{1}}{E_{3}}=\frac{A_{1}/l}{V_{3}/t}$$

where *S_1_* represents the strain along the 1st direction, measured as the ratio between the vibration amplitude, *A_1_* and the plate’s length, *l*, and *E_3_* represents the electric field along the 3rd direction, calculated as the ratio of applied voltage, *V_3_,* to the thickness, *t*. The LDV was set up as shown in Figure 11a, with a Mn:PIN-PMN-PT LTE plate mounted on a spring-loaded electrode fixture and the beam focused on the side of the LTE plate.

An AC signal of constant 5 V amplitude was swept spectrally across the LTE resonance from 100 to 200 kHz. The vibration amplitude, *V_3_* and current are shown in Figure 11b. *V_3_* dropped and the current increased at resonance because of the low electric impedance; the vibration amplitude increase approximately matched the increase in current. Applying Equation (5) off resonance, *d_31_* = 457 pC/N was measured at 100 kHz for this sample, a value similar to the figure of 474 pC/N provided by the supplier (TRS Technologies, State College, PA, USA).

To compare the LDV measurement with different methodologies, a study was conducted with Mn:PIN-PMN-PT samples with different vibration modes, as shown in Table 4. All the measurements were performed locally under ambient conditions, except for the material supplier’s data. There is reasonable agreement between the different techniques, with no single technique consistently appearing to over or underestimate the coefficient values. Equally, however, this data set offers no gold standard measurement techniques in close agreement with each other.

**Table 4:** Characterisation of piezoelectric constant *d_ij_* on Mn:PIN-PMN-PT under ambient conditions with different vibration modes measured with different techniques.

(pC/N)	Mode	Berlincourt Method	IEEE Method	LDV	Manufacturer Data
*d_33_*	LE	1053	1492	1160	-
	TE	1062	-	-	-
*d_31_*	LTE	478	458	402	467
*d_31_^45z∆^*	LTE	518	493	457	474
*d_15_*	TS	156	120	156	121
*d_36_*	FS	-	-	1109	1128

*d_31_^45z∆^*: *d_31_* value for LTE sample rotated 45° about *z* axis [12].

## 5. Discussion

Table 5 summarises the basic temperature and pressure characteristics of the three generations of piezocrystal as considered in this paper. Where comparison is possible with the literature, it is generally in good agreement, except for a much higher figure for *T_RT_* for Mn:PIN-PMN-PT, as measured approximately in the present work. In consideration of high power transducer operation, this suggests that Generation II and III piezocrystals (PIN-PMN-PT and Mn:PIN-PMN-PT respectively) have usefully raised temperature limits. However, material suppliers may care to look into the issue of phase transitions under the influence of pressure for widest acceptance of piezocrystals.

**Table 5:** Basic comparison between three generations of piezocrystals.

Crystal	*T_C_*^ ◊^ (°C)	*T_C_* * (°C)	*T_RT_*^ ◊^ (°C)	*T_RT_* * (°C)
PMN-PT	135	≈130	96	≈100
PIN-PMN-PT	191	>180	125	≈130
Mn:PIN-PMN-PT	193	>180	119	≈150
**Crystal**	**σ*_RO_*^ ◊^ (MPa)**	σ*_RO_* * (MPa)	***E_c_*^ ◊^ (kV/cm)**	***E_int_*^ ◊^ (kV/cm)**
PMN-PT	≈15 MPa	≈10 MPa	2.3	0.0
PIN-PMN-PT	≈20 MPa	≈20 MPa	5.0	0.0
Mn:PIN-PMN-PT	>21 MPa	>20 MPa	6.0	1.0

^◊^ Literature values [1,28,34,40,41]; * Measured values in this study.

**Figure 12:**
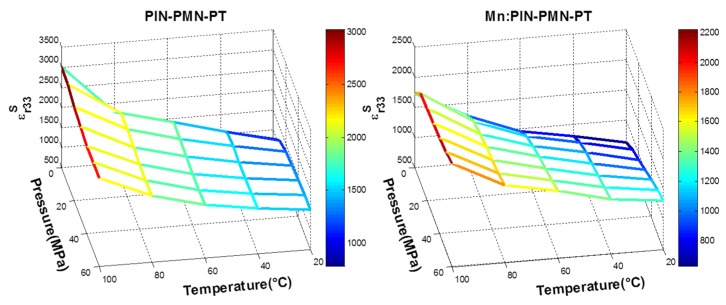

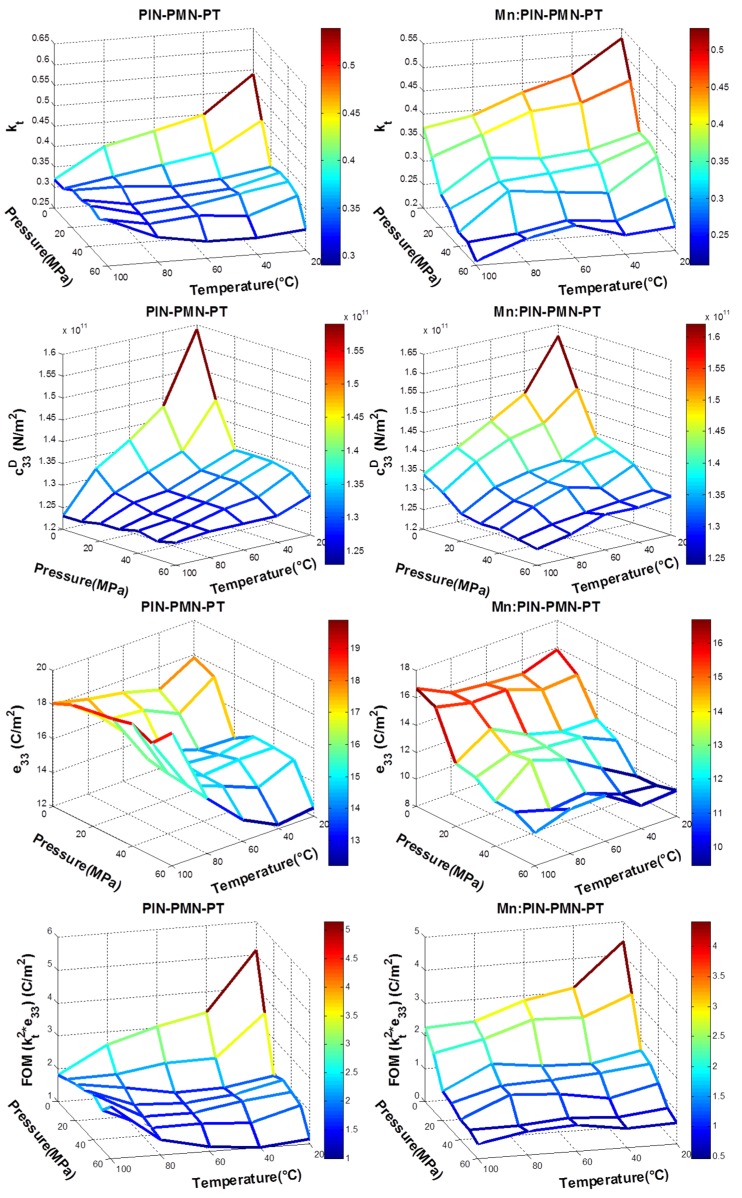
A summary of the variation of key parameters of PIN-PMN-PT and Mn:PIN-PMN-PT with varying temperature and pressure on ε*^S^_r33_*, *k_t_*, *c^D^_33_*, *e_33_* and one figure of merit (*k_t_^2^e_33_*).

Figure 12 summarises the variation in the key material parameters with temperature and pressure, presented in a format to allow easier interpretation and comparison. As expected from many previous reports in the literature, ε*^S^_r33_* increases with *T*; however, changes in *P* have less effect. Electromechanical coupling, *k_t_*, and stiffness, *c^D^_33_*, are significantly higher at ambient *T* and *P*, and they generally fall as either and both of these parameters increases. In both cases, the reduction is likely to be sufficient to require a device designer to take it into account if the operating envelope of the device approaches the limits of *T* and *P* considered here. The most complicated characteristics are exhibited by *e_33_*. Mn:PIN-PMN-PT shows relatively little change with *T* but a generally falling trend with *P*. PIN-PMN-PT shows similar characteristics up to 60 °C then a recovery to the ambient temperature values for all values of *P*. This may be caused by a phase transition mediated by *T* and *P*. However, further work is required to confirm this.

The FOM, *k_t_^2^e_33_*, is finally shown in Figure 12 for PIN-PMN-PT and Mn:PIN-PMN-PT and the values are detailed in Table 6a,b respectively, with the addition of variances calculated from ambient to peak *T* and *P*. Somewhat contrary to expectation, this suggests that PIN-PMN-PT generally exhibits less variance in FOM than Mn:PIN-PMN-PT, crucially at a combination of the highest values of *T* and *P*. We believe this tends to support our proposal that a phase transition is responsible for the variation under the extreme of the conditions considered here. It is also notable that the values for FOM reduce very significantly as *T* and *P* increase.

**Table 6:** (**a**) Detail of FOM *k_t_^2^e_33_* (C/m^2^) for PIN-PMN-PT with variation in *T* and *P*; (**b**) Detail of FOM *k_t_^2^e_33_* (C/m^2^) for Mn:PIN-PMN-PT with variation in *T* and *P*.

**(a)**								
***T*/*P***	**0 MPa**	**10 MPa**	**20 MPa**	**30 MPa**	**40 MPa**	**50 MPa**	**60 MPa**	**Variation**
20 °C	5.16	3.52	1.89	2.07	1.99	1.88	1.15	−77.8%
40 °C	3.36	2.26	1.65	1.74	1.82	1.46	1.00	−70.3%
60 °C	3.04	2.23	1.62	1.59	1.58	1.56	1.14	−62.4%
80 °C	2.62	2.01	1.89	1.67	1.60	1.55	1.48	−43.5%
100 °C	1.80	1.96	2.12	2.24	2.39	2.20	2.60	44.7%
**Variation**	−65.1%	−44.4%	12.2%	8.3%	20.6%	17.1%	127.2%	−**49.5%**
**(b)**								
***T*/*P***	**0 MPa**	**10 MPa**	**20 MPa**	**30 MPa**	**40 MPa**	**50 MPa**	**60 MPa**	**Variation**
20 °C	4.42	3.09	1.67	1.56	1.24	0.80	0.64	−85.6%
40 °C	3.12	2.50	1.38	1.39	0.91	0.62	0.57	−81.6%
60 °C	2.84	2.56	1.44	1.29	1.16	0.73	0.80	−71.9%
80 °C	2.32	2.08	1.62	1.25	1.26	0.60	0.72	−68.9%
100 °C	2.25	1.77	0.89	0.82	0.62	0.60	0.47	−79.1%
**Variation**	−49.2%	−42.7%	−46.6%	−47.5%	−50.0%	−25.0%	−26.3%	−**89.4%**

As summarised in Table 5 and Table 6, deterioration in piezocrystal functional performance under a combination of temperature, stress and electric field has been noted and has remained of research interest since investigations started in PMN-PT. Early characterisation was done on LE and LTE modes with a series of *T* and *P* values applied and phase transitions were examined to explain the performance variations. A satisfactory dimensional height:width:depth ratio was suggested as 3:1:1 to define a LE mode bar for the application of pressure [40,41]. Following that work, with the development of PIN-PMN-PT, characterisation was done under conditions of elevated *T*, *P* and *E* with special attention focused on the stress-induced ferroelectric rhombohedral (*F_R_*) to ferroelectric orthorhombic (*F_O_*) phase transition [28]. The relevant value, σ = 20 MPa, is consistent with the measurements in our study. Another study was reported more recently, including the newly developed Mn:PIN-PMN-PT, with uniaxial pressure and different electric fields, and reductions in the piezoelectric constant, *d_32_*, were demonstrated in both PIN-PMN-PT and Mn:PIN-PMN-PT [34]. The results reported here add to the evidence of such performance reductions and reinforce the need for functional characterisation of new piezocrystal materials to allow device designers to estimate the effects of their behaviour on practical performance.

## 6. Conclusions and Further Work

The use of piezocrystals to exploit their potential high performance brings with it the need to consider performance variations contingent on both their growth processes and their intrinsic behavior. This paper has reported application-oriented research to determine trends in the latter when the three generations of these materials are subject to ranges of temperature, pressure and drive voltages (electric fields) representative of practical use. This research has been based on a measurement system including an oven to apply elevated temperatures and a material testing system for the application of uniaxial pressure, as shown in Figure 2, for measuring the passive response of the piezocrystals to their environment. Through high drive components, the system also allows the response to active transducer driving to be determined, e.g., in terms of increases in temperature because of drive conditions. Furthermore, it allows comparison of different measurement methods, such as electrical impedance spectroscopy and laser Doppler vibrometry.

Using this system, it has been shown that Generation I, PMN-PT, is relatively sensitive to temperature, though with marginally the highest peak performance under ambient conditions. Given the low coercive field values reported in the literature, this suggests it is most suitable for applications demanding low average power levels and with low excitation fields to avoid the need for DC bias. This matches practical experience, with PMN-PT now as the material of choice for biomedical imaging. Generation II, PIN-PMN-PT, and Generation III, Mn:PIN-PMN-PT, materials have been developed specifically for applications with greater demands in terms of temperature and pressure, such as are typically found in power ultrasound, including SONAR and the oil and gas industry. Here, we have confirmed that samples of both generations have higher phase transition and Curie temperatures than Generation I PMN-PT. However, pressure in the range to 60 MPa appears to have a highly deleterious effect on parameters such as TE mode coupling coefficient, *k_t_*, and Figure of Merit, FOM = *k_t_^2^e_33_*. In addition, our specific measurements suggest relatively little or no advantage from Mn-doping in terms of immunity to environmental temperature and pressure. However, we have not considered coercive field, *E_c_*, or mechanical quality factor, *Q_m_*, in detail, and both of these parameters have been reported to be significantly higher and useful for performance assessment in active transduction.

In considering work to further elucidate the behaviour of piezocrystals for practical configurations, we noted that the temperature investigation presented in Section 4.1 ranged upwards from 20 °C. However, it may be useful to determine the behaviour at lower temperatures for applications such as underwater SONAR. Although commercial temperature-controlled cryostats can provide very wide temperature ranges and environmental chambers are available, their configurations are generally unsuitable for simultaneous application of pressure in the range up to 60 MPa, representing full ocean depth. Initial trials of low temperature characterisation were carried out in our lab based on the system described in this paper, cooling the load applicator bars in a −80 °C freezer and then assembling them with further cooling packs to allow characterisation to start at 0 °C. Further investigation of the use of LN_2_ is under consideration to make this arrangement less cumbrous.

Another issue that has been considered in a preliminary way is the effect of pressure cycling and fatigue. Measurements were performed on PIN-PMN-PT and Mn:PIN-PMN-PT; comparison with a hard piezoceramic is also of interest. For both piezocrystals, property variations are particularly evident for 0 ≤ *P* ≤ 25 MPa; after only three pressure cycles, *k_t_* was found to have suffered a permanent reduction in both materials while the properties of the hard ceramic were much more stable under loading and there was no long term change upon completion of a small number of pressure cycles. This clearly merits further consideration.

Benefitting from the increase in computational power owing to Moore’s law, FEA has largely superseded analytical methods for commercial transducer design. This technique helps to verify design validity but generally requires the full elastoelectric matrix as input data. Measurement of this matrix under varying conditions of temperature, pressure and electric field allows the transducer to be simulated under a range of operating conditions, in principle. However, whilst measurement is feasible over a range of temperatures, it is difficult at elevated pressure because of the likelihood of mechanical damage, especially for the LE mode bar. In addition, the generation of a high electric field may be difficult because the thickness of this sample demands a very high applied voltage. In this context, techniques similar to those reported by Cao *et al*. may be helpful, using the elastoelectric matrix of a known piezoelectric material as an initial condition and applying optimisation techniques to reduce the number of samples required to obtain the matrix for the new material. Additional techniques combining simulation and modelling, such as those reported by Gallagher and Lynch to characterise piezocrystal properties across the phase transition, can reduce the number of measurements and combination of external conditions needed to fully characterise the material [44]. Such techniques are also beginning to find favour in commercial FEA packages, and it is expected that these will be combined with the external application of temperature and pressure in due course.
